# Gait style during weave pole performance affects limb dynamics in agility dogs

**DOI:** 10.1002/vro2.70028

**Published:** 2026-02-25

**Authors:** Charlotte Ramsey, Roberta Blake

**Affiliations:** ^1^ School of Agriculture, Animal and Environmental Sciences Anglia Ruskin University Writtle UK

## Abstract

**Background:**

Canine agility is a physically demanding sport that carries an inherent risk of injury. The weave pole obstacle is a mandatory component in agility courses under UK Kennel Club regulations, requiring a complex forward and lateral side‐to‐side gait that is not typically replicated outside the sport. This study aimed to evaluate key kinetic parameters—peak force (PF), peak vertical force (PVF), vertical impulse (VI) and stance time (ST)—experienced by forelimbs and hindlimbs during weave pole performance, and to assess differences across the three most common forelimb gait variations: front‐feet single‐stepping rear double (FFSS/RD), double‐stepping (FFDS) and hopping (FFH).

**Methods:**

Seventeen experienced agility dogs completed a set of six competition‐standard weave poles (FFSS/RD, *n* = 8; FFDS, *n* = 4; FFH, *n* = 5). Data were collected using two pressure‐sensing walkways and statistically analysed.

**Results:**

Outer limbs, in general, have shown significantly higher PVF, PV, VI and ST (*p* < 0.001 for all variables on both forelimbs and hindlimbs). Peak vertical force was significantly greater in the outer forelimbs and outer hindlimbs of FFH dogs compared to FFDS and FFSS/RD (*p* < 0.001 and 0.021, respectively, for forelimbs; *p* = 0.014 and 0.003, respectively, for hindlimbs). Stance time and VI were higher on the outer forelimb on FFSS/RD (the only forelimb used in this style) in comparison with FFH (ST: *p* = 0.003; VI: *p* = 0.024) and FFDS (ST: *p* = 0.041; VI: *p* = 0.032).

**Conclusions:**

Overall, there is a clear asymmetry in outer and inner limbs in all styles, which is consistent to the expected on turns. All gaits have shown a trend on redistribution of load towards the outer hindlimb, which was more extreme on FFSS/RD. Stance time was generally lower, and PVF and PF were higher in the FFH group. These factors raise questions regarding the long‐term implications of weave pole performance in dogs exhibiting this gait.

## INTRODUCTION

Canine agility is a high‐intensity sport in which canine athletes are directed through a predetermined sequence of 15‒20 obstacles—including weaves, tunnels, hurdles and contact obstacles—with those completing the course in the fastest times without incurring faults through knocked poles, skipped contacts or refusals earning podium positions. The weave task has been a component of a standard Kennel Club agility course since the origin of sports in 1978,^1^ with current UK Kennel Club regulations stating that this obstacle must appear once within a standard agility or jumping course as either a set of six or 12, with the dog entering the obstacle with the first pole adjacent to the dogs left shoulder.^2^ The current 600 mm spacing was introduced in 2012 to aid larger dogs in comfortably completing the obstacle,^3^ and to ensure the obstacle stability, the support base of the poles is set up with the support legs protruding in the opposite direction to the dog's path of travel.

To date, biomechanical research on weave pole performance remains scarce compared to obstacles such as the dog walk,^4^ A‐frame^5^, ^6^, ^7^, ^8^, ^9^, ^10^ and single bar jump,^11^, ^12^, ^13^, ^14^, ^15^, ^16^, ^17^ which have received increasing attention in studies investigating biomechanical demands and mechanisms of injury. This focus likely stems from retrospective findings, which consistently highlight these obstacles as more commonly associated with injury (17.5%, 15.9%, and 36.5%, respectively) according to Inkilä et al.,^18^ compared to the weave poles, which have a reported injury rate of between 6.3% and 13.9%.^18^, ^19^

Despite the evident complexity of the weave task, no research has yet objectively evaluated the biomechanical demands of this task. A considerable degree of lateral spinal flexion and shoulder abduction/adduction is required to allow a large dog to manoeuvre through the poles. With many of the large championship dogs completing a full set of 12 poles in less than 3 s, these changes in kinematics are occurring rapidly, thus presenting a significant opportunity for injuries to arise, emphasising the need for further investigation into the biomechanical demands and potential injury risks associated with weave pole performance.

It is also particularly difficult to draw inferences from other gait patterns or movements performed outside of agility because the unique forward and lateral plane side‐to‐side motion is not typically replicated by any other quadruped outside of the sport.^20^ Preliminary research on weave pole performance by Eicher et al.^20^ analysing videos of 1377 dogs of 53 different breeds identified that all dogs could be separated into five distinct gait patterns (Table 1), finding that the three most common forelimb gait variations are: front‐feet single‐stepping rear double (FFSS/RD), double‐stepping (FFDS) and hopping (FFH). This preliminary research forms a foundation for further biomechanical research; however, the reasons for gait preference are believed to be multifactorial, and the implications of each gait on body biomechanics and longevity remain poorly understood.

**TABLE 1:** Gait pattern variations identified by Eicher et al.^20^

Gait pattern	Definition
Front‐feet single‐stepping, rear feet single	Dog lands on the lateral forelimb paw only, pushing off with the lateral forelimb paw only. Medial forelimb paw does not make contact with the ground between the weave poles. Rear feet single step.
Front‐feet single‐stepping, rear feet double	Dog lands on the lateral forelimb paw only, pushing off with the lateral forelimb paw only. Medial forelimb paw does not make contact with the ground between the weave poles. Rear feet double step using both hindlimbs.
Front‐feet double‐stepping	Dog lands using both forelimbs, but the lateral paw makes initial contact, followed by the medial paw, pushing off with both feet between poles.
Front‐feet hopping	Both paws land simultaneously, with the dog pushing off with both feet simultaneously between poles.
Multiple stepping/walking	No clear paw placement pattern or competitor walks through the poles.

Nevertheless, given the disconcertingly high prevalence of shoulder and spinal injuries reported across all available literature, and the visible, repetitive demands of this obstacle on both the spine and shoulders of canine athletes, it is of high importance that the demands of this obstacle are understood and further investigation is warranted. A detailed exploration of both kinetic and kinematic demands could yield valuable insights into potential injury mechanisms, thereby informing strategies for modifying training practices or obstacle design to mitigate injury risk. Although recent adjustments to other agility obstacles have not significantly reduced injury rates, a comprehensive examination of the biomechanics of weave pole performance may prove critical in understanding and addressing shoulder injuries in canine athletes. Therefore, this study aimed to investigate the peak force (PF), peak vertical force (PVF), vertical impulse (VI) and stance time (ST) experienced by the forelimbs and hindlimbs during weave pole performance, and how these vary between the three most common gait styles: FFSS/RD, FFH and FFDS.

## MATERIALS AND METHODS

All dogs were physically sound and in good health prior to the trial and were monitored throughout for signs of stress, illness, injury or fatigue. To minimise injury risk, each dog was given an adequate warm‐up and habituation period, as well as access to breaks and fresh drinking water. None of the participants required more than five attempts to achieve the valid number of passes for the trial. If any dogs required more than five attempts, they would have been asked to take a 10‐min break before retrying and if any dogs required more than 10 attempts, they would have been excluded on ethical grounds.

### Sample population

Upon completion of the resource equation,^21^ it was determined that a minimum of five dogs would be required within this research. Dog and handler partnerships were recruited through relevant Facebook groups, and following recruitment, 17 dogs were recruited, with six dogs each in the FFSS/RD and FFH groups and five dogs in FFDS group, according to owner self‐report. However, upon kinematics and kinetics analysis, the dogs have been correctly classified within gait patterns as described by Eicher et al.^20^, and the final sample, after gait pattern correct identification, consisted of eight dogs in the FFSS/RD group, five dogs in the FFH group, and four dogs in the FFDS group. While some dogs utilised more than one pattern—noticeably when entering and exiting the last pole—dogs were grouped by dominant gait style through the middle weave poles. These dogs were of various breeds and sizes, aged 5.94 ± 2.49 years, and weighed 17.4 ± 7.44 kg (mean ± standard deviation). The inclusion criteria of this study stated that the dogs had to be at competition level, over 2 years of age, with a minimum of 1 year of regular training or competition experience. The subject demographics can be seen by group in Table 2.

**TABLE 2:** Subject demographics per group after correction.

Dominant gait style	Weight (kg)	Breed	Age (years)
FFDS	12.7	Springer X Min Poodle	5
	10.9	Cocker Spaniel	4
	17.8	Miniature American Shepherd	4
	15.1	Miniature American Shepherd	2
Mean ± SD	14.13 ± 2.59		3.75 ± 1.258
FFSS/RD	33.1	Weimaraner	7
	16.6	Border Collie	11
	17.7	Border Collie	3
	16.8	Border Collie	7
	24.6	German Shorthaired Pointer	6
	27.2	Weimaraner	5
	29.2	Weimaraner	8
	22.6	Lurcher X	7
Mean ± SD	23.48 ± 5.77		6.75 ± 2.315
FFH	7.0	Bichon X	7
	7.4	Bichon X	9
	9.9	Cocker Spaniel	9
	10.8	Cocker Spaniel	4
	16.4	Border Collie	3
Mean ± SD	10.30 ± 3.38		6.40 ± 2.793

Abbreviations: FFDS, front‐feet double‐stepping; FFH, front‐feet hopping; FFSS/RD, front‐feet single‐stepping rear double.

### Experimental set‐up

**FIGURE 1:**
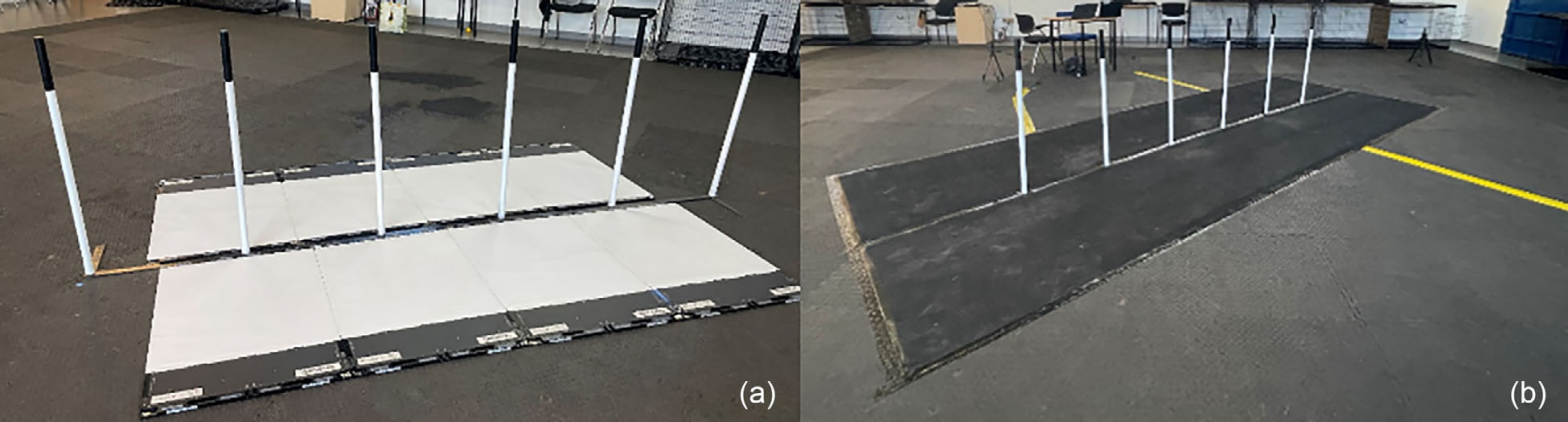
Pressure mapping system tiles in arrangement over the legs of the weave pole base (a), and full trial set‐up with tiles covered by a rubber mat (b).

A set of six plastic weave poles conforming with both the UK Kennel Club and Federation Cynologique International regulations (Callieway, Austria) were set up according to the current regulations (5 m distance between obstacle and starting point, support base legs protruding in the opposite direction to the dog's path of travel)^3^ in the secure indoor training venue. Once situated, four standard sensitivity Strideway version 4 pressure plates (Tekscan) were arranged sequentially in a longitudinal way on either side of the weave poles, creating two separate platforms each consisting of four plates (Figure 1a).

To ensure smooth entry and exit from the obstacle, ascent and descent ramps were then fixed to either end of the platforms, and 5‐mm‐thick medium‐high‐density foam strips (600 mm × 40 mm) were placed in the gaps where the Strideway platforms met the weave poles to create a seamless surface.

Following the pressure‐sensing system assembly, two rolls of 5 m × 1 m, 3‐mm‐thick non‐slip rubber matting were secured over the Strideway system with heavy duty tape, both around the edges—securing the matting to the floor and at the seam where the two separate sections of matting meet between the poles. Holes were cut to allow the screw mechanism for each pole to protrude through the rubber without disrupting the surface, and the poles were then reattached over the top, further securing the rubber matting in place. Most of the force platforms wires could be safely directed out of the trial zone to the required plug sockets and two data collection laptops. However, where this was not possible, high‐visibility, heavy duty tape was used to secure the wires to the floor to ensure that these were not a trip hazard for either the human or canine participants. The starting point was measured and marked out with tape at 5 m from the first weave pole. This final set‐up can be seen in Figure 1b.

### Kinetic data collection

The Strideway system was used to collect values for inner and outer forelimb and hindlimb PF, PVF, VI and ST. This system contained 0.968 sensels/cm^2^ collected at 500 Hz (Strideway, Tekscan) and was calibrated before commencing data collection according to manufacturer's instructions. The left and right systems remained separate, with data for each Strideway platform (left and right sides) being collected on a separate laptop. The active sensing area of each individual platform measured 0.65 m × 2.6 m, resulting in a total sensing area beneath the poles of 1.3 m × 2.6 m.

### Video collection

Two cameras (iPhone, Apple) were mounted on tripods, with one being positioned perpendicular to the weave poles so that all six poles were within the frame. The second camera was positioned 3.5 m away from the final weave pole, capturing footage of the trial area from a cranial view. A video was captured (240 fps) and synchronised with the kinetic data obtained to allow a comparison of the data to be made with the visual gait patterns observed.

### Data analysis

For each dog, three valid passes of the weave poles were required, with passes being retained when the dog completed the poles entering from the correct start point into the correct side, completing all six poles without skipping poles, or exiting and returning to the obstacle. Data collected adjacent to pole three (the centre) during each valid pass were averaged and analysed to generate representative values for each dog.

Kinetic data were normalised by bodyweight in Newtons to enable comparisons to be made between dogs of various sizes, and analysed by proprietary software (Tekscan Walkway, v.7.02), with these kinetic parameters consisting of individual forelimbs and hindlimbs (PVF, PF and VI). Peak vertical force was defined as the maximum vertical ground reaction force measured during each footfall. Peak force was considered the highest area of force on each footfall, not the total area of contact, but simply the area of the highest force within a stance pawprint. Vertical impulse, defined as the integral of the vertical ground reaction force over the ST (impulse = force × time), was averaged across all limb strikes for each limb and is reported in common standard units of N s.

Furthermore, two kinematic variables were also analysed to support the kinetics data. Stance time was defined as the elapsed time, in seconds, of contact with the ground of the limb in question. Speed was measured in metres per second from the videos and was a product of the weaves total length (3 m) by the time the dog took to complete them.^21^ Load distribution (%) on each gait pattern was defined by limb PVF/total limbs PVF.

### Statistical analysis

All analyses were performed using jamovi (version 2.6.44) (The jamovi project, 2025) using the GAMLj module. All the results are presented as plots to provide a visual representation of the data. The data are shown as mean ± standard deviation, unless otherwise stated. A linear mixed model (LMM) was used as it is a commonly used statistical test in canine biomechanical research.^17^ For the LMM, the data were divided into two subsets, one containing all the parameters for forelimbs and another containing all the parameters for the hindlimbs. To analyse the prediction of parameter variance by the categorical descriptors of the gait pattern, non‐significant effects were reduced one‐by‐one, starting with the complete model until the final reduced model satisfied a minimal Akaike information criterion. First, backward random effects elimination was performed, followed by elimination of the fixed effects. *p*‐Values were calculated using Satterthwaite's approximation for degrees of freedom. The LMMs were used to investigate the impact of ‘gait patterns’ (FFH, FFDS and FFSS/RD) and the ‘limb’ (inner/outer) on PVF, PF, VI and ST. The full model considered the interactions gait pattern × limb. ‘Dog’, which contained the individuals, was set as a random effect. ‘Speed’ was set as covariate as it could have affected the kinetics parameters, so we wanted to test this correlation. All models were fitted using restricted maximum likelihood estimation. Model acquirements and assumptions were fulfilled, as variances were homogeneous and residuals were normally distributed. Post hoc analyses of main effects and interaction effects were carried out using comparison of least mean squares of group means with Bonferroni correction. Load distribution was analysed with two‐way ANOVA (Welch's), with Games‒Howell post hoc tests with on between subject (gait pattern) and one within‐subject factor (limb). The significance level was defined for a *p*‐value of 0.05 or less.

## RESULTS

All the animals completed the study, with no adverse effects. The FFSS/RD gait is excluded from inner forelimb results as it does not involve an inner limb placement. The mean ± standard deviation of all variables can be seen in Supporting Information S1.

An overview of the main effects and interactions of ‘gait pattern’ and ‘limb’, as well as the covariance caused by the random effect and the covariate (speed) effect, is shown in Table 3.

**TABLE 3:** Main effects and interactions of final reduced linear mixed models of variables obtained during experiment to investigate the influence of gait patterns on limb dynamics variables during agility weaves performance, by comparing dogs performing three different gait patterns: front‐feet single‐stepping rear double, front‐feet hopping and front‐feet double‐stepping.

	Forelimb PVF	Forelimb PF	Forelimb VI	Forelimb ST	Hindlimb PVF	Hindlimb PF	Hindlimb VI	Hindlimb ST
Random effect								
Dog	9.46%	4.06%	9.46%	1.09%	5.21%	0.94%	4.68%	1.37%
Covariate effect								
Speed	*p* = 0.420	*p* = 0.981	*p* = 0.068	*p* = 0.037	*p* = 0.262	*p* = 0.929	*p* = 0.147	*p* = 0.186
Fixed effects								
Gait pattern	*p* < 0.001^a^	*p* < 0.001^a^	*p* = 0.011	*p* = 0.002^a^	*p* = 0.002^a^	*p* < 0.001	*p* = 0.558	*p* = 0.223
Limb (inner vs. outer)	*p* < 0.001^a^	*p* < 0.001^a^	*p* < 0.001	*p* < 0.001^a^	*p* < 0.001^a^	*p* < 0.001	*p* < 0.001	*p* < 0.001
Interactions effects								
Gait pattern × limb	*p* < 0.001	*p* = 0.006	*p* = 0.320	*p* < 0.001	*p* = 0.024	*p* = 0.288	NA	NA

*Note*: Random effect (dog) values are shown as individual variance (%). *p* > 0.05 fields indicate the effect was not included in the final model. The final reduced model for each parameter can be obtained from the non‐empty fields in the corresponding table column. For example, the final reduced model for forelimb PVF contains gait pattern and limb as main effects, gait pattern × limb as interaction effect.

Abbreviations: PF, peak force; PVF, peak vertical force; ST, stance time; VI, vertical impulse.

^a^Interaction‐driven main effects.

**FIGURE 2:**
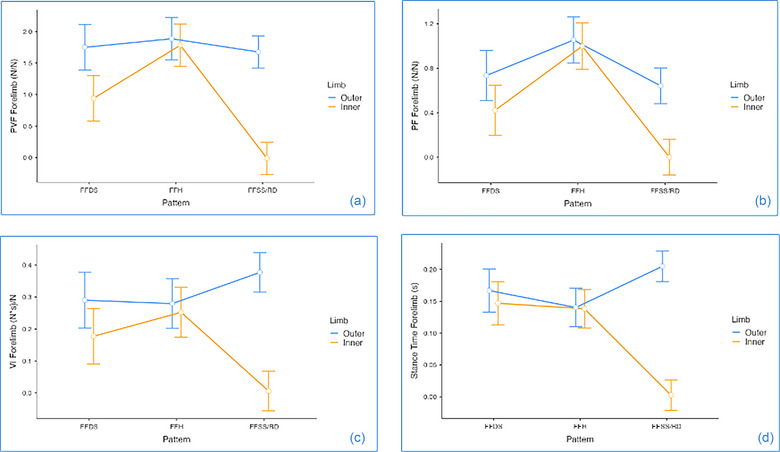
Interaction plots for main effects of gait and inner versus outer limbs on: (a) peak vertical force (PVF), (b) peak force (PF), (c) vertical impulse (VI) and (d) stance time (ST) on forelimbs for dogs performing agility weave poles utilising front‐feet single‐stepping rear double (FFSS/RD) (*n* = 8), front‐feet hopping (FFH) (*n* = 5) and front‐feet double‐stepping (FFDS) (*n* = 4) gait patterns. Forces and impulses are normalised to bodyweight for each dog to correct for variation between dogs. Points show mean values. Error bars show standard errors around the means.

**FIGURE 3:**
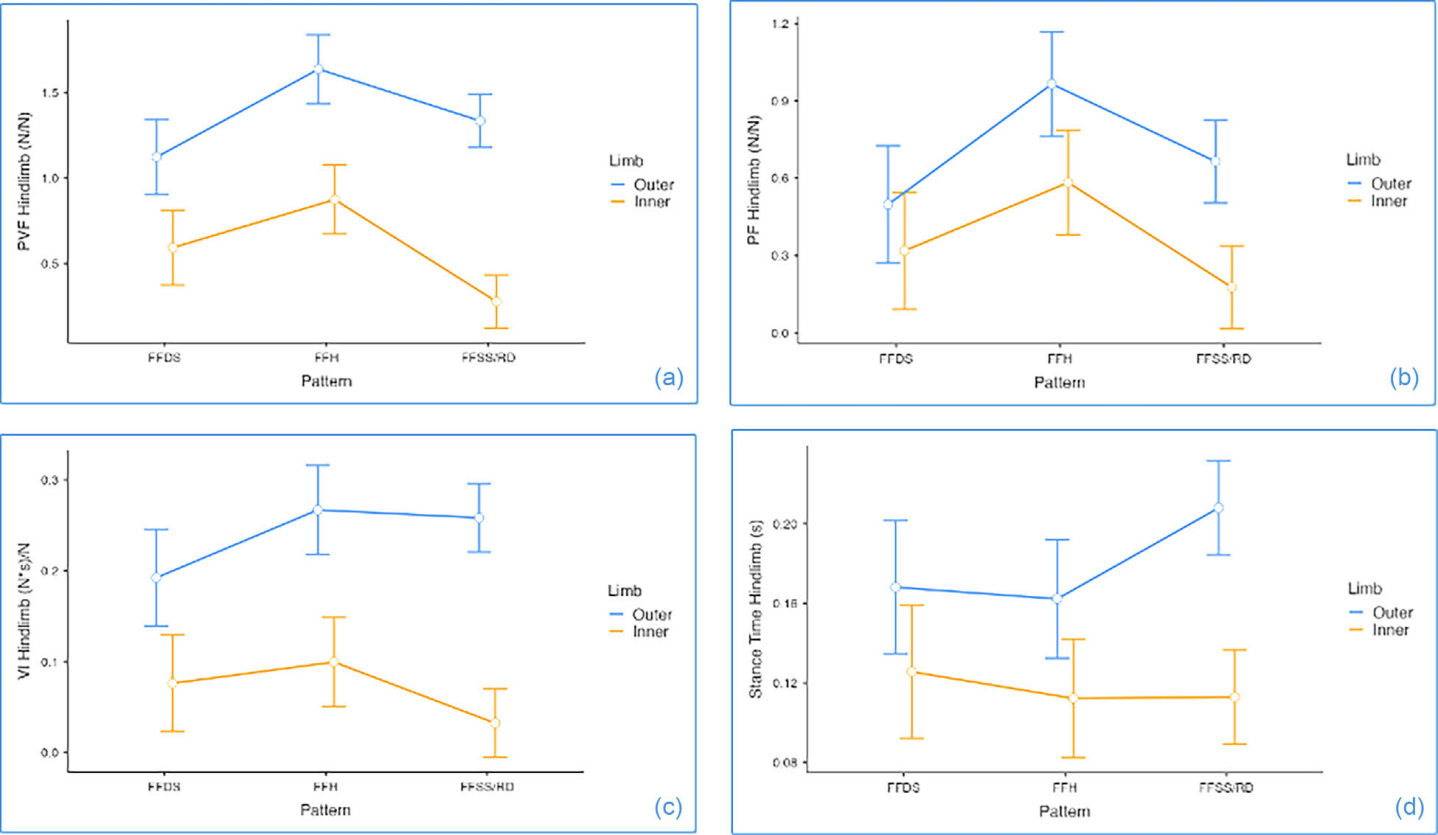
Interaction plots for main effects of gait and inner versus outer limbs on: (a) peak vertical force (PVF), (b) peak force (PF), (c) vertical impulse (VI) and (d) stance time (ST) on hindlimbs for dogs performing agility weave poles utilising front‐feet single‐stepping rear double (FFSS/RD) (*n* = 8), front‐feet hopping (FFH) (*n* = 5) and front‐feet double‐stepping (FFDS) (*n* = 4) gait patterns. Forces and impulses are normalised to bodyweight for each dog to correct for variation between dogs. Points show mean values. Error bars show standard errors around the means.

Speed, as covariate effect, has shown influence only on the forelimb ST (*p* = 0.037) and has shown no effect on the kinetic variables. Most of the main effects were interaction driven and are reported with asterisks in Table 3. Although, it is acknowledged that interaction‐driven main effects do not necessarily need to be reported, they are described for clarity. Figure 2 shows the LLM main effect results for forelimbs and Figure 3 shows the main effects for the hindlimbs.

### Gait pattern main effect

For forelimbs, PVF had an effect of gait, with the pattern FFH showing significant higher PVFs than FFDS and FFSS/DS (*p *< 0.001 and 0.021, respectively). Similarly, for hindlimbs, FFH has shown a higher PVF, when compared with the other two patters, FFDS (*p* = 0.014) and FFSS/RD (*p* = 0.003). Front‐feet hopping has shown higher PF than FFDS and FFSS/DS on both forelimb (*p* = 0.004 and 0.001, respectively) and hindlimb (*p* = 0.005 and 0.002, respectively). Stance time and VI on outer forelimb were higher on the FFSS/RD than FFH (ST: *p* = 0.003; VI: *p* = 0.024) and FFDS (ST: *p* = 0.041; VI: *p* = 0.032).

### Limb main effect

For all variables analysed (PVF, PF, VI and ST), the outer limb has shown significantly main effect, with higher values for forelimbs (*p* < 0.001) and hindlimbs (*p* < 0.001) for all the variables.

### Interaction effect gait pattern × limb

Inner forelimb analysis for FFSS/RD was not included. We observed four significant interactions gait pattern × limb in the forelimbs (PVF, PF, ST and VI) and only one in the hindlimbs (PVF). We have not reported the pairwise significant interaction effects, which were driven only by the main effect (e.g., FFH outer > FFH inner), which have already been reported above. For the forelimbs, when the main effects of gait pattern and limb have been excluded, PVF has shown a significant interaction effects, particularly with the outer forelimb on FFH having higher PVF than FFDS inner forelimb (*p* = 0.008) and, therefore, being the forelimb with higher PVF among all interactions. Furthermore, the outer forelimb on FFSS/RD had higher PVF than the FFDS inner forelimb (*p* = 0.033). For the hindlimbs, more interaction effects were seen for PVF, with FFH showing higher PVF on the outer hindlimb in comparison to the inner hindlimbs from the other patterns (*p* < 0.001, for both) and therefore being the hindlimb with higher PVF overall. Front‐feet hopping has also shown higher PVF on the inner hindlimb in comparison with the FFSS/RD outer hindlimb. Additionally, FFSS/RD outer hindlimb had higher PVF than FFDS inner hind (*p *< 0.001). When PF was looked, FFH outer forelimb had higher PF than then inner limbs on FFDS (*p* = 0.004), with outer FFH showing high PF overall. A last interaction effect was identified for PF, where the FFDS outer forelimb had higher PF then the FFSS/RD inner forelimb. For the interaction effect of gait pattern × limb for VI on forelimbs, the FFSS/RD outer forelimb was higher than FFDS inner (*p* = 0.011).

### PVF percentage distribution between limbs within each gait pattern

**FIGURE 4:**
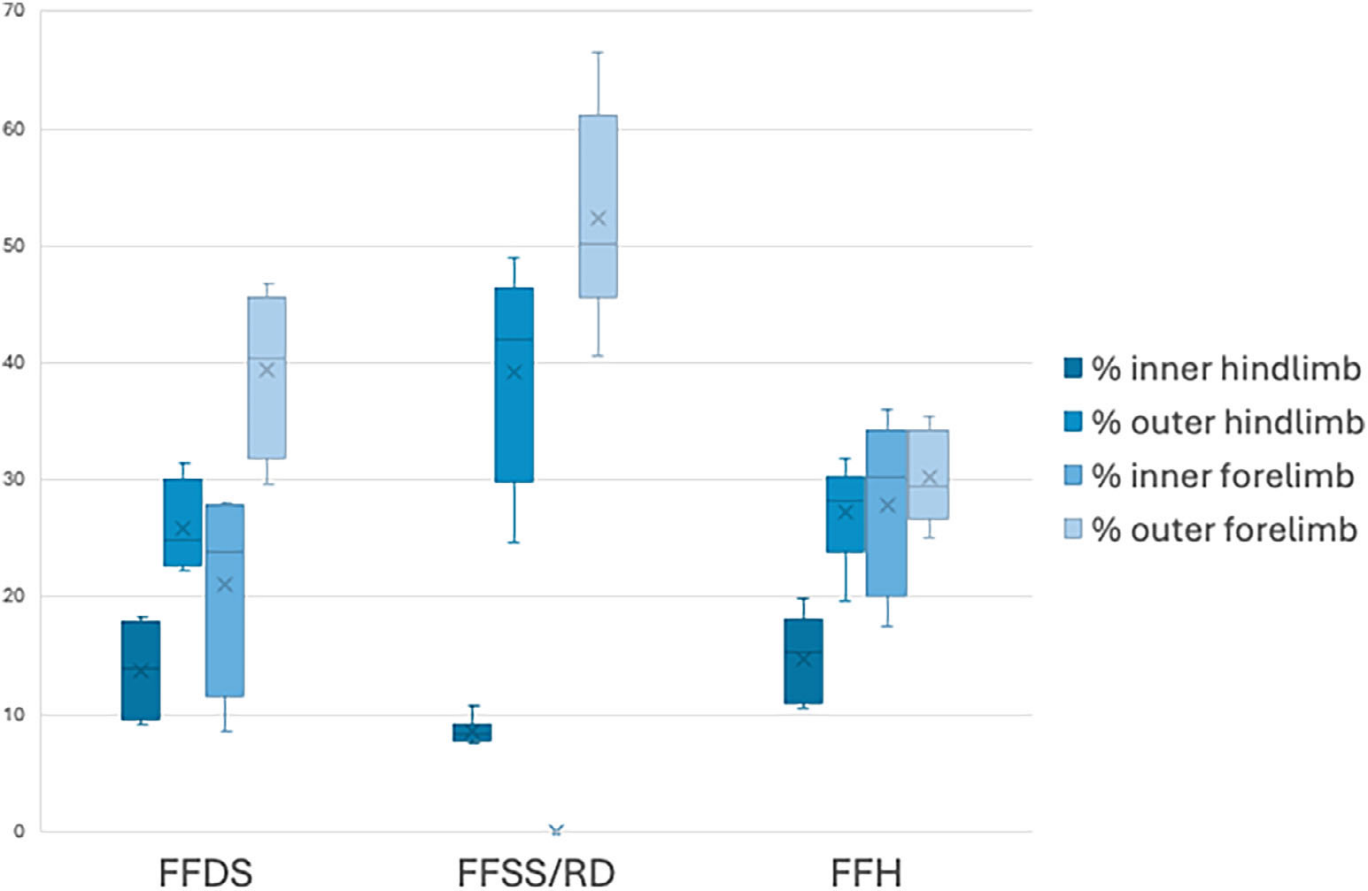
Load distribution (%) during each one of the gait patterns for dogs performing agility weave poles utilising front‐feet single‐stepping rear double (FFSS/RD) (*n* = 8), front‐feet hopping (FFH) (*n* = 5) and front‐feet double‐stepping (FFDS) (*n* = 4) gait patterns. The bottom and top of the box are the first and third quartiles, the band inside the box is the second quartile (the median), and ‘x’ is the mean. The lines extending vertically from the boxes indicate the minimum and maximum of all the data.

The overall FFH has shown a better uniform distribution of load among the four limbs, with the outer hindlimb receiving a load distribution similar to that of the forelimbs, and lower load on the inner hindlimb (*p* = 0.049). The FFSS/RD had, for both outer forelimbs (*p* < 0.001) and outer hindlimbs (*p* = 0.023), extremely high load (%) in comparison with the inner limbs and with the other patterns. Conversely, it has shown a very low value of load distribution on the inner hindlimb (*p* = 0.003) (and obviously none on inner forelimb). The FFDS had higher loads on the outer hindlimbs and forelimbs in comparison with the inner limbs, with outer hindlimb load exceeding the inner forelimb load (*p* = 0.012) (Figure 4).

## DISCUSSION

The aim of this research was to assess whether differences in gait patterns within the weave poles resulted in significant variation on limb dynamics parameters, including ST, VI, PVF and PF. This was assessed using a sample of 17 healthy, sound, competition‐level agility dogs, with *n* = 4 utilising the FFDS pattern, *n* = 8 using the FFSS/RD pattern, and *n* = 5 using the FFH pattern. Each dog completed three valid passes through a set of six UK Kennel Club specification weave poles, during which kinetic data were collected using a series of pressure mapping plates.

The results of this research somewhat support the acceptance of the alternate hypothesis of this study as significant effects of gait patterns were identified for PF, PVF and forelimb ST. Furthermore, limb effects (either inner or outer limb) were significant for all variables. Speed has not affected most of the variables, showing an effect only on forelimb ST. Overall, outer limbs have shown higher PVF, PF, VI and ST in both forelimbs and hindlimbs. Particularly, FFH outer limbs have shown significantly higher PVF and PF overall, despite having the most uniform load distribution (%) among the four limbs. Front‐feet single‐stepping rear double and FFDS have shown outer hindlimbs with higher load distribution than inner forelimbs, showing a possible shift in centre of mass (CoM) in these patterns, diagonally towards outer hindlimbs.

This research highlights several interrelated points to consider when analysing the biomechanical demands of each gait pattern. It is not yet fully understood why dogs opt to utilise one gait pattern through the weave poles over another, although significant differences in selection by height/breed have been observed previously through the weave obstacle by Eicher et al.^20^ Therefore, further supporting size and conformation are contributing factors. According to Eicher et al.,^20^ smaller dogs competing in the 16‐in height class were identified as much more likely to utilise the FFH gait than taller dogs competing in the 20‒24‐in height class, which were more likely to utilise FFDS or FFSS/RD gaits. These findings are largely consistent with observations in this research (based on the assumption that larger mass is related to greater height—a characteristic common to all dogs included in this study), whereby the largest dogs by mass (23.48 ± 5.77 kg) opted for the FFSS/RD gait, with the smallest mass group (10.30 ± 3.88 kg) opting to use the FFH gait.

Both the FFDS and FFH gaits utilise both forelimbs to some degree within each stride. However, the FFSS/RD gait is unique in that it only utilises the outer forelimb within the stride, with the inside forelimb instead ‘swimming’ through without making contact in preparation to land on the next stride. On the FFSS/RD pattern, the inner hindlimb shows a minimal load distribution (8.57 ± 1.00%), with Eicher et al.^20^ identifying this gait as yielding a significantly faster completion time (hypothetically due to a shorter limb contact time), this raises the concern that the outer limbs are experiencing all of the force, relative to the FFDS and FFH gaits, where this force is distributed between all limbs. Considering only the outer limbs, FFH still exceeds the PVF measured in outer forelimbs and hindlimbs in comparison with the other gaits. However, the FFSS/RD pattern has greater asymmetry in load between inner and outer limbs, with indications of shifting on CoM towards the outer hindlimbs, as seen on the FFDS pattern as well.

Another factor that is particularly pertinent to note is that one forelimb contact, alongside the faster completion times, would anecdotally render the forelimbs at a greater risk of injury in this gait, as the dog's ability to distribute the force over a longer period of time would be reduced. This could pose an increased risk for injuries, unless the animal takes other mitigating action to lessen the forces on this limb, such as shifting the centre of mass over the hindlimbs or altering their limb joint biomechanics when landing on a single leg, as identified in human research comparing single‐limb landing to double‐limb landing.^22^

Although not significant or directly comparable due to methodological differences, the results from this research produced findings that differ from the hypotheses of previous literature. Front‐feet single‐stepping rear double dogs in this research instead yielded longer outer forelimb STs relative to those in the FFDS and FFH groups allowing these dogs a longer period of contact allowing time to distribute the forces, explaining the lower PVF on FFSS/RD in comparison with other gaits, which may occur, or be a result of a multitude of interrelated factors. Despite having a low PVF, the FFSS/RD has shown the highest VI on forelimbs because the extended ST, which uses only one forelimb requires a higher impulse generation to maintain forward movement.

The path of travel through the weave poles could be conceptualised as a series of alternating elongated semicircles. Anecdotally, for smaller dogs, these semicircles would be more elongated with a smaller radius, as they do not have to generate as much lateral flexion to move between the weave poles and can adopt a straighter trajectory. This is due in part to the fact that unlike other obstacles, the distances between weave poles are not adapted relative to height category. Conversely, larger dogs with longer spines will have to generate significantly greater lateral flexion relative to smaller dogs in order to pass between the 60 cm weave pole spacing. With some having a head‐to‐pelvis length that exceeds the distance between weave poles, this would necessitate a wider arc with a larger radius to accommodate and generate this lateral flexion.

Inner‒outer limb force asymmetries on a turn have been noted across a wide variety of both bipedal and quadruped literature,^23^, ^24^, ^25^, ^26^ which explains the clear outer/inner limb asymmetries identified during weave negotiation. Söhnel et al.^16^ assessing canine turning manoeuvres following a hurdle further concluded that the outside abducted forelimb is responsible for generating turning power on a turn and exhibits higher forces and higher impulses than the inside limb, which provides the required stability. Furthermore, Blake and Blake^27^ have observed considerable kinematic asymmetries in outer and inner limbs in flyball dogs during the box turn, while Blake et al.^28^ have identified higher PVF on outer hindlimbs on flyball dogs during the box turn.

As the FFSS/RD gait does not utilise an inner limb placement, the outer forelimb during the weave obstacle is responsible for both the maintenance of stability and turning power generation. Thus, the outer forelimb allows for the generation of a stable force generation, and has an increased ST, as highlighted in Figure 2, which aids in generating the lateral spinal flexion and redirection of the body while also providing greater control and stability. This principle may therefore explain the increased ST on this limb relative to the other FFDS and the FFH gaits. The FFSS/RD outer limb experiences a greater ST and VI relative to the inside limb to generate the turn, with the inside limb instead appearing to have a stabilising/pivot function.^16^

Despite the FFSS/RD gait only using one forelimb, relative to FFDS and FFH, which distribute the weight over both forelimbs, FFDS and FFH have shown higher PVF on forelimbs. This can be explained that, relative to their height, dogs performing FFSS/RD are negotiating the obstacle in a lower dimensionless speed than their counterparts. The dimensionless speed normalises absolute speed by accounting for differences in withers height, thus enabling fair comparisons between dogs.^29^ This therefore suggests that each gait pattern is adapted in such a way that it circumvents differences in limb placements and prevents a detrimental increase in PVF.

**FIGURE 5:**
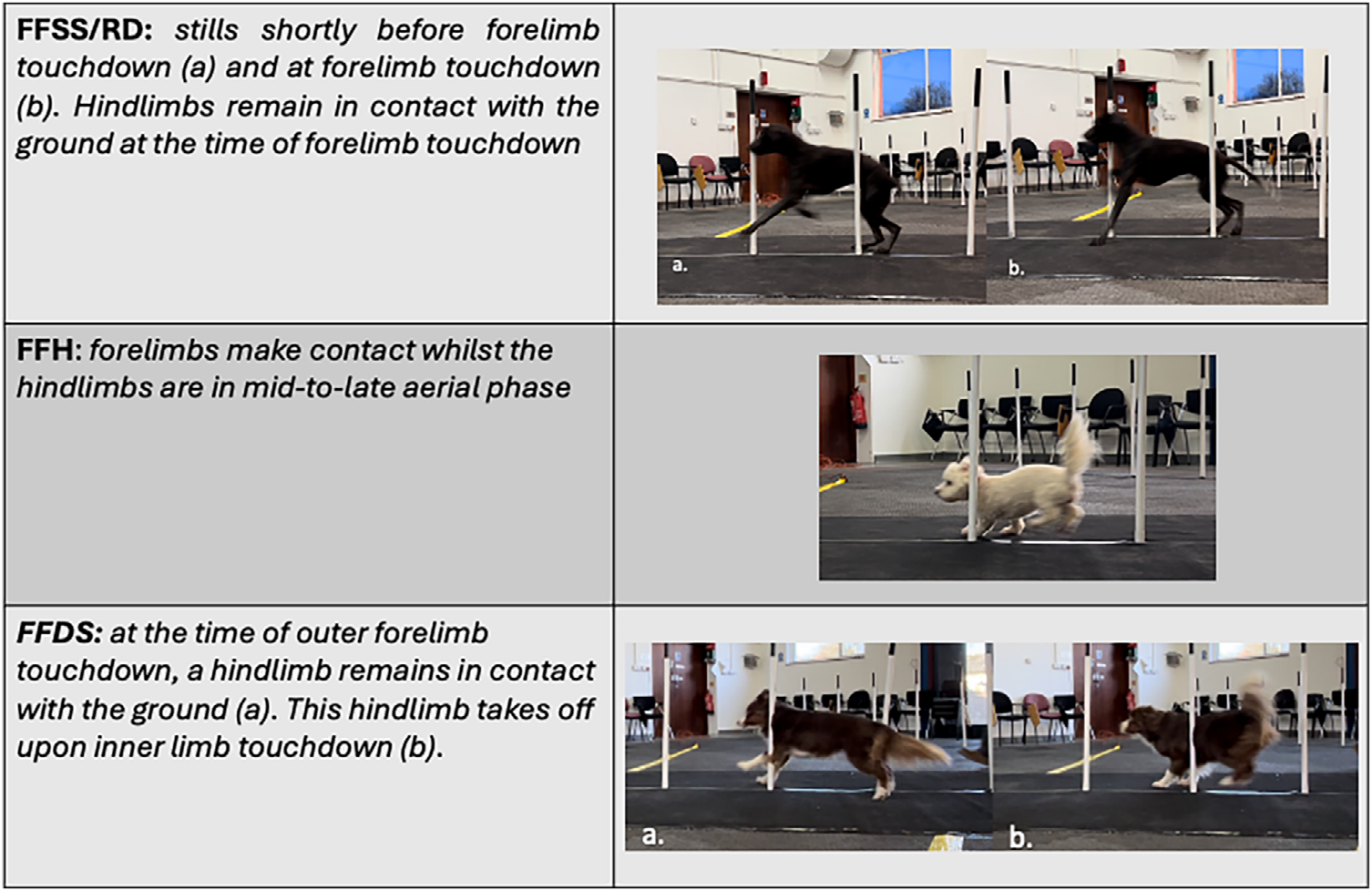
Stills of dogs performing front‐feet single‐stepping rear double (FFSS/RD), hopping (FFH) and double‐stepping (FFDS) styles.

To date, to the authors knowledge, aside from identification of gait preferences, no further analysis has been conducted on how the body mass is distributed during the weave pole obstacle or how forelimb placement is timed relative to hindlimb placement. This research has, however, begun to elucidate such factors, highlighting potential mechanisms for force reduction within the FFSS/RD gait, such as increasing ST, while FFH and FFDS have shown higher forces associated with the obstacle. When observing obtained footage of the FFSS/RD gait dogs from a lateral view, at the time of forelimb touchdown, the hindlimbs were still in stance, with the dog's weight appearing to be shifted over the hindlimbs. The mechanisms by which different dogs achieved this varied slightly. Most dogs appeared to work in a more collected gait, with an obviously caudal shift of weight over the hindlimbs and good hindlimb tracking, with the hindlimbs landing where the forelimbs take off (Figure 5). However, the Border Collies tended to opt for a more crouched posture, although still maintaining good tracking of the hindlimbs. This more collected movement allows the dog to navigate between the poles while reducing bodyweight distribution over the forelimbs, maintaining stability, and increasing ST necessary when only one forelimb is used on a stride and the CoM is shifted to the outer limbs. Additionally, with increasing ST, PVF is reduced,^30^, ^31^ as the body can distribute the forces over a longer period of time,^14^ thus further reducing the PVF experienced by the individual forelimb of this gait.

Conversely, given that the current definition of the FFH group is ‘both forelimb paws lad simultaneously and push off simultaneously between poles’,^20^ it would be expected that these dogs have a more even force distribution, with relatively lower values for PVF, as the forces are distributed across two forelimbs. However, again, the findings from this research differ to earlier findings by Eicher et al.^20^ Although there was less asymmetry in the timings of the inner‒outer forelimb placement compared to FFDS, only two of the dogs participating in this trial under the FFH group actually made simultaneous contact with the forelimbs, despite appearing to the eye to land in synchronisation. This difference was only apparent when analysing kinetic data from the force plates, therefore highlighting the limitations of earlier literature in defining gait patterns, as these primary observations were made by the human eye, which can only see process images at around 77 fps.^32^ However, we have seen that although hopping has higher PVF and PF, probably due to shorter ST, this pattern is the one with least asymmetries between outer and inner limbs, probably due to the nearly synchronous landing of the pair of limbs.

Interestingly, even in the absence of synchronous forelimb contact, FFH was still distinct when compared to the FFDS gait, in that at the time of forelimb touchdown, on FFH the hindlimbs were in their mid‐to‐late aerial phase, with all of the FFH dogs jumping onto the forelimbs, as displayed in Figure 5. Conversely, in FFDS, at the time of outer forelimb touchdown, there was at least one hindlimb instance, with the hindlimbs entering the aerial phase upon inner forelimb touchdown, as shown in Figure 5. This is likely a mechanism to increase the length of their stride, as relative to the bigger dogs, the smaller dogs have more ground to cover between weave poles.^33^ This, combined with the shorter ST that the FFH dogs exhibit as displayed, raises concern for the high forces in the forelimbs and hindlimbs by action of hopping and apparent shift of the CoM towards the hindlimbs and outer limbs.

**FIGURE 6:**
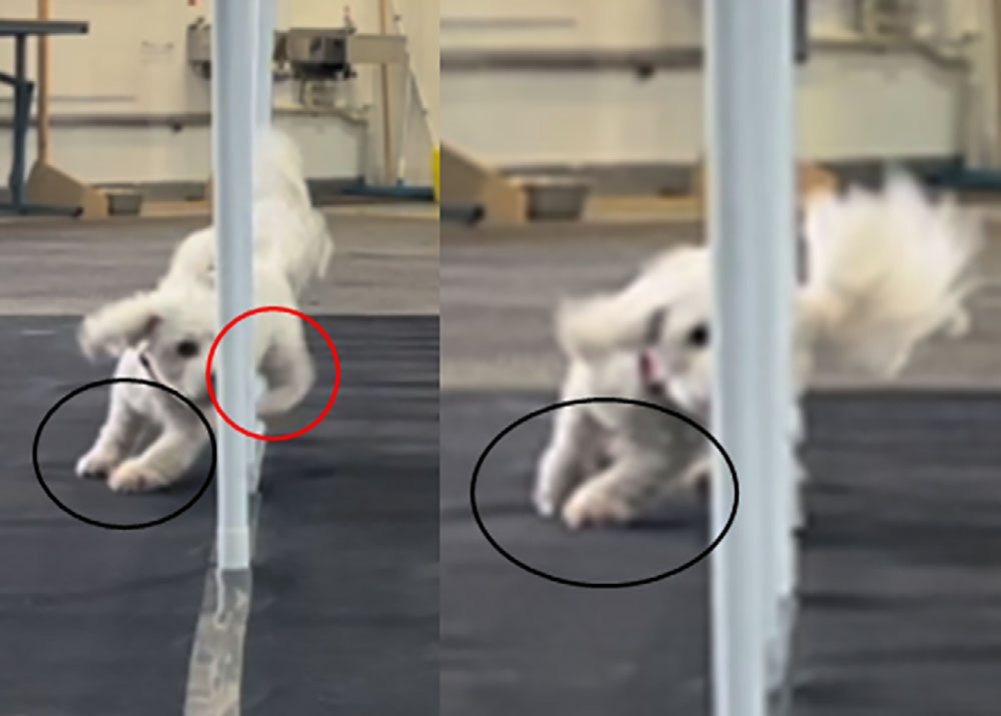
Cranial view of participant from front‐feet hopping group. Note that the inner forelimb is positioned in front of the outside forelimb and makes contact while the hindlimbs are still aerial. The dog then moves over this forelimb while it is still in stance, appearing to adduct and compress the limb.

One difference in these dogs is that the FFH group is the only group to experience similar PVF and PF in the inner limbs relative to the outer limbs, thus suggesting that in these dogs, the inner and outer limbs work in nearly synchronous movement, possibly distributing the load more evenly than in the FFDS and FFSS/RD groups, where the outer limbs clearly exceed in PVF and PF in relation to inner limbs. The inner limb of the FFH dogs was typically loaded slightly first and positioned in front of the outside limb, with the dog beginning to move back over this limb while it was still in stance, as presented in Figure 6. This observation was noted across the smaller dogs opting to use the FFH gait, with the one larger dog of the group being an outlier to this pattern. This dog was also one of the younger dogs of the cohort, suggesting that this dog may not yet have settled into a consistent pattern. This may also be again, related to the degree of the turn required as smaller dogs can take a straighter path, thus not requiring as much impulse to turn the body back in the allocated direction; however, in the absence of any other larger dogs in this group, this observation is not fully understood.

The final observation to discuss from this research is that the most consistent findings among all gaits are the outer/inner limb asymmetries, such as those reported in other turn studies,^16^, ^27^, ^28^ and this asymmetry is particularly more evident on the hindlimbs, maybe due to their propulsive role. However, it is important to note that unlike flyball dogs, where dogs turn only to one side, weaves negotiation involves equal turns to the right and left, which would ensure a balance on the forces involved.

Although appropriate measures were taken to minimise the impact of potential limitations during this trial, some unavoidable constraints remained and should be considered when interpreting the findings of this research.

In line with previous research, kinetic parameters were normalised to bodyweight to account for inter‐individual differences in size, as canine morphology has been identified as the greatest source of variance within heterogeneous populations in previous canine research.^34^ While this approach reduces variance, it does not eliminate it entirely. Furthermore, this method has previously been considered insufficient for accounting for differences in body size when evaluating time‐dependent variables in earlier canine research by Bertram et al.,^35^ as smaller dogs must travel at a relatively higher velocity than large dogs to cover the same distance over the same time, possibly explaining the increased PVF and PF on FFH, which is performed by smaller dogs. Velocity is believed to influence ground reaction forces,^31^ and unlike other canine gait studies using controlled velocity conditions, this is not possible when studying agility obstacles given that the weave poles are an obstacle completed off lead without handler input, the dog self‐selects its velocity, and this cannot be externally controlled. Although we have not identified difference in speeds between the styles, this may be due to dog's size differences and the same absolute speed for high dogs is likely to be less impactful than for a smaller dog.

Another limitation lies in the sampling method used for this research. The sample for this research was based on convenience, with local volunteers being recruited through online advertisement. As gait preferences were seemingly linked to dog size, this necessitated the use of a heterogenous sample, spanning multiple breeds, ages and sizes, which enhances the ecological validity of the study and better reflects the diversity seen in the canine agility community. On the other hand, it introduces variability that could obscure subtle biomechanical trends or confound interpretation.

There is also the added potential for training methods to act as a confounder, with dogs of the same family potentially all being taught using the same method, which may introduce bias. Retrospective research has previously highlighted that the two‐by‐two weave method is associated with a significantly greater risk of severe injury,^36^ although the mechanisms behind this observation are not fully understood. Given that this method requires more repetition to shape the desired behaviour than the channel or V‐weave method, it is plausible that this repetition increases the dog's predisposition to overuse and strain injuries. However, there is also potential for different weave training methods to influence gait preference and body biomechanics, and this relationship has not been investigated. These training‐related factors were not controlled for in the present study and warrant further investigation.

Unfortunately, sample size became a limitation of this research as, during the data collection, when the gait styles were checked, two of the attending dogs performed different gaits to the one the owner had initially identified. This left the FFDS group a dog short of the minimum number required to satisfy the resource equation. Therefore, the subject demographic Table 2 reflects the gait styles and groupings used for analysis after correction for gait identification, not the patterns self‐reported by the owner during recruitment.

Finally, the absence of the measurement of dimensionless speed, due to the lack of dogs’ height data, has not allowed a normalised speed to be compared between patterns.

From this research, each gait pattern appears to incorporate biomechanical strategies to assist in its completion. For instance, in FFSS/RD dogs, CoM appears to be distributed caudally to lessen the force travelling through one forelimb, or in FFH dogs jumping onto the forelimbs, CoM appears to land for the most part, with two feet simultaneously or near simultaneously to more evenly distribute the forces through the forelimbs and hindlimbs, thus suggesting that these adaptive strategies suggest that different gait patterns may serve functional roles in managing mechanical stress.

Further research should focus on investigating and quantifying the kinematic parameters of each weave pole pattern, investigating whether there is any interplay between joint kinematics and kinetics. Differences in lateral spinal flexion and shoulder abduction/adduction between different gait patterns and sizes may also contribute to injury development, especially when considered alongside differences in ground reaction forces. Understanding these relationships could enhance veterinary understanding of the mechanisms underlying common injuries in agility dogs and inform preventive strategies.

Further kinetic trials such as this one, including a larger sample size and a full set of 12 poles embedded in a more realistic set‐up, may aid in more reliably confirming the significance of observed trends, and mitigate the effects of confounders and outliers. Longitudinal studies tracking performance and injury outcomes in dogs with different weave pole gait patterns or weave‐pole‐focused questionnaires for dogs that have previously sustained an injury attributed to weave pole performance could also assist in identifying patters of injury in real‐life scenarios to form hypotheses for future kinematic and kinetic research. Ultimately, combining more focused retrospective research and primary biomechanical research will be key to elucidating the potential mechanisms of weave‐pole‐related injuries or the obstacles contribution to common pathologies, and will be instrumental in addressing a significant gap in the current literature. Last, research has investigated whether reducing the pole distance for smaller dogs would trigger a change in gait from FFH to FFDS or FFSS, which in theory would be safer.

## CONCLUSION

Peak vertical force and PF were significantly greater in the FFH group compared to the FFSS/RD and FFDS groups. The outer forelimb ST and VI was higher on the FFSS/RD, which in turn resulted in lower forces acting on these limbs. With all these factors combined, this raises further questions as to the long‐term implications of weave pole performance on the dogs performing this gait and thus is certainly an area requiring further research.

This is currently the only known study investigating the kinetic effects of weave pole performance, and whether there is any difference in parameters between the three dominant forelimb gait styles. This research acts as a preliminary investigation into the kinetic demands of the weave poles and provides insight into the forces experienced by the forelimbs and how these differ between gait preferences, while also contributing to a broader understanding of gait classification patterns beyond forelimb placement alone.

## AUTHOR CONTRIBUTIONS

*Conceptualisation, methodology, investigation, formal analysis and writing—original draft preparation*: Charlotte Ramsey. *Formal analysis, supervision, resources, visualisation and writing—review and editing*: Roberta Blake.

## CONFLICTS OF INTEREST

The authors declare they have no conflicts of interest.

## FUNDING INFORMATION

This research received no grants or funding from any funding agency in the public, commercial or not‐for‐profit sectors.

## ETHICS STATEMENT

The authors confirm that the ethical policies of the journal, as noted on the journal's author guidelines page, have been adhered to. The research was approved by the Ethics Research Panel of Anglia Ruskin University, with approval number ETH2425‐1215. Owners were provided with an information sheet detailing the trial and provided full written consent for each dog prior to the commencement of the trial.

## Supporting information



Supporting Information

Supporting Information

## Data Availability

The data that support the findings of this study are openly available at Anglia Ruskin Research Online at https://figshare.com/s/704a88d63d1ad4143c7a.
