# Supplementary material for: Gait style during weave pole performance affects limb dynamics in agility dogs
Source: Vet Rec Open. 2026 Feb 25;13(1):e70028. doi: 10.1002/vro2.70028 (PMC12935563; doi:10.1002/vro2.70028)
Supplement: Supplementary file 1 — Supporting Information [file VRO2-13-e70028-s002.docx]

Supplementary file S1

Table S1. Mean and standard deviation of all variables (*peak vertical force (PVF), peak force (PF), vertical impulse (VI), stance time (ST) and speed)* for the completion of six weaves of dogs performing front-feet single-stepping, rear double (FFSS/RD), front-feet hopping (FFH), and front-feet double-stepping (FFDS), for forelimbs and hindlimbs*.*

|  | **Pattern** | **Limb** | **Mean** | **SD** |
| --- | --- | --- | --- | --- |
| **PVF Forelimb (N/N)** | **FFDS** | **Outer** | 1.7367 | 0.36360 |
|  |  | **Inner** | 0.9274 | 0.39224 |
|  | **FFH** | **Outer** | 1.8360 | 0.30447 |
|  |  | **Inner** | 1.7335 | 0.64364 |
|  | **FFSS/RD** | **Outer** | 1.6880 | 0.27715 |
|  |  | **Inner** | 0.0000 | 0.00000 |
| **PVF Hindlimb (N/N)** | **FFDS** | **Outer** | 1.1349 | 0.15646 |
|  |  | **Inner** | 0.6053 | 0.18911 |
|  | **FFH** | **Outer** | 1.6448 | 0.19125 |
|  |  | **Inner** | 0.8820 | 0.19701 |
|  | **FFSS/RD** | **Outer** | 1.3257 | 0.33512 |
|  |  | **Inner** | 0.2694 | 0.02918 |
| **PF Forelimb (N/N)** | **FFDS** | **Outer** | 0.7349 | 0.26728 |
|  |  | **Inner** | 0.4222 | 0.10979 |
|  | **FFH** | **Outer** | 1.0294 | 0.24739 |
|  |  | **Inner** | 0.9743 | 0.43630 |
|  | **FFSS/RD** | **Outer** | 0.6422 | 0.08878 |
|  |  | **Inner** | 0.0000 | 0.00000 |
| **PF Hindlimb (N/N)** | **FFDS** | **Outer** | 0.4974 | 0.07423 |
|  |  | **Inner** | 0.3175 | 0.05870 |
|  | **FFH** | **Outer** | 0.9652 | 0.39488 |
|  |  | **Inner** | 0.5829 | 0.16042 |
|  | **FFSS/RD** | **Outer** | 0.6656 | 0.28314 |
|  |  | **Inner** | 0.1770 | 0.01665 |
| **VI Forelimb (N*s)/N** | **FFDS** | **Outer** | 0.2972 | 0.04447 |
|  |  | **Inner** | 0.1842 | 0.10063 |
|  | **FFH** | **Outer** | 0.2833 | 0.04994 |
|  |  | **Inner** | 0.2561 | 0.04986 |
|  | **FFSS/RD** | **Outer** | 0.3716 | 0.15305 |
|  |  | **Inner** | 0.0000 | 0.00000 |
| **VI Hindlimb (N*s)/N** | **FFDS** | **Outer** | 0.1944 | 0.04322 |
|  |  | **Inner** | 0.0783 | 0.02896 |
|  | **FFH** | **Outer** | 0.2705 | 0.06579 |
|  |  | **Inner** | 0.1032 | 0.04419 |
|  | **FFSS/RD** | **Outer** | 0.2567 | 0.07515 |
|  |  | **Inner** | 0.0308 | 0.01018 |
| **Stance Time Forelimb (s)** | **FFDS** | **Outer** | 0.1700 | 0.04082 |
|  |  | **Inner** | 0.1500 | 0.02944 |
|  | **FFH** | **Outer** | 0.1420 | 0.03564 |
|  |  | **Inner** | 0.1400 | 0.03391 |
|  | **FFSS/RD** | **Outer** | 0.2025 | 0.04979 |
|  |  | **Inner** | 0.0000 | 0.00000 |
| **Stance Time Hindlimb (s)** | **FFDS** | **Outer** | 0.1700 | 0.01414 |
|  |  | **Inner** | 0.1275 | 0.00957 |
|  | **FFH** | **Outer** | 0.1633 | 0.02626 |
|  |  | **Inner** | 0.1133 | 0.04024 |
|  | **FFSS/RD** | **Outer** | 0.2064 | 0.04050 |
|  |  | **Inner** | 0.1114 | 0.03563 |
| **Speed (m/s)** | **FFDS** | **Outer** | 2.0107 | 0.27814 |
|  |  | **Inner** | 2.0107 | 0.27814 |
|  | **FFH** | **Outer** | 2.0410 | 0.31419 |
|  |  | **Inner** | 2.0410 | 0.31419 |
|  | **FFSS/RD** | **Outer** | 2.1310 | 0.28136 |
|  |  | **Inner** | 2.1310 | 0.28136 |
