# Supplementary material for: Gait style during weave pole performance affects limb dynamics in agility dogs
Source: Vet Rec Open. 2026 Feb 25;13(1):e70028. doi: 10.1002/vro2.70028 (PMC12935563; doi:10.1002/vro2.70028)
Supplement: Supplementary file 2 — Supporting Information [file VRO2-13-e70028-s001.pdf]

Supplementary file S1

Table S1. Mean and standard deviation of all variables (*peak vertical force (PVF), peak force (PF), vertical impulse (VI), stance time (ST) and speed)* for the completion of six weaves of dogs performing front-feet single-stepping, rear double (FFSS/RD), front-feet hopping (FFH), and front-feet double-stepping (FFDS), for forelimbs and hindlimbs.

	Pattern	Limb	Mean	SD
**PVF Forelimb (N/N)**	**FFDS**	**Outer**	1.7367	0.36360
		**Inner**	0.9274	0.39224
	**FFH**	**Outer**	1.8360	0.30447
		**Inner**	1.7335	0.64364
	**FFSS/RD**	**Outer**	1.6880	0.27715
		**Inner**	0.0000	0.00000
**PVF Hindlimb (N/N)**	**FFDS**	**Outer**	1.1349	0.15646
		**Inner**	0.6053	0.18911
	**FFH**	**Outer**	1.6448	0.19125
		**Inner**	0.8820	0.19701
	**FFSS/RD**	**Outer**	1.3257	0.33512
		**Inner**	0.2694	0.02918
**PF Forelimb (N/N)**	**FFDS**	**Outer**	0.7349	0.26728
		**Inner**	0.4222	0.10979
	**FFH**	**Outer**	1.0294	0.24739
		**Inner**	0.9743	0.43630
	**FFSS/RD**	**Outer**	0.6422	0.08878
		**Inner**	0.0000	0.00000
**PF Hindlimb (N/N)**	**FFDS**	**Outer**	0.4974	0.07423
		**Inner**	0.3175	0.05870
	**FFH**	**Outer**	0.9652	0.39488
		**Inner**	0.5829	0.16042
	**FFSS/RD**	**Outer**	0.6656	0.28314
		**Inner**	0.1770	0.01665
**VI Forelimb (N*s)/N**	**FFDS**	**Outer**	0.2972	0.04447
		**Inner**	0.1842	0.10063
	**FFH**	**Outer**	0.2833	0.04994
		**Inner**	0.2561	0.04986
	**FFSS/RD**	**Outer**	0.3716	0.15305
		**Inner**	0.0000	0.00000

	Pattern	Limb	Mean	SD
**VI Hindlimb (N*s)/N**	**FFDS**	**Outer**	0.1944	0.04322
		**Inner**	0.0783	0.02896
	**FFH**	**Outer**	0.2705	0.06579
		**Inner**	0.1032	0.04419
	**FFSS/RD**	**Outer**	0.2567	0.07515
		**Inner**	0.0308	0.01018
**Stance Time Forelimb (s)**	**FFDS**	**Outer**	0.1700	0.04082
		**Inner**	0.1500	0.02944
	**FFH**	**Outer**	0.1420	0.03564
		**Inner**	0.1400	0.03391
	**FFSS/RD**	**Outer**	0.2025	0.04979
		**Inner**	0.0000	0.00000
**Stance Time Hindlimb (s)**	**FFDS**	**Outer**	0.1700	0.01414
		**Inner**	0.1275	0.00957
	**FFH**	**Outer**	0.1633	0.02626
		**Inner**	0.1133	0.04024
	**FFSS/RD**	**Outer**	0.2064	0.04050
		**Inner**	0.1114	0.03563
**Speed (m/s)**	**FFDS**	**Outer**	2.0107	0.27814
		**Inner**	2.0107	0.27814
	**FFH**	**Outer**	2.0410	0.31419
		**Inner**	2.0410	0.31419
	**FFSS/RD**	**Outer**	2.1310	0.28136
		**Inner**	2.1310	0.28136
